# The galactose elimination capacity and mortality in 781 Danish patients with newly-diagnosed liver cirrhosis: a cohort study

**DOI:** 10.1186/1471-230X-9-50

**Published:** 2009-06-30

**Authors:** Peter Jepsen, Hendrik Vilstrup, Peter Ott, Susanne Keiding, Per K Andersen, Niels Tygstrup

**Affiliations:** 1 Department of Clinical Epidemiology, Aarhus University Hospital, Aarhus, Denmark; 2 Department of Medicine V (Hepatology and Gastroenterology), Aarhus University Hospital, Aarhus, Denmark; 3 Department of Biostatistics, Institute of Public Health, University of Copenhagen, Copenhagen, Denmark; 4 Department of Medicine A, Rigshospitalet, Copenhagen, Denmark

## Abstract

**Background:**

Despite its biologic plausibility, the association between liver function and mortality of patients with chronic liver disease is not well supported by data. Therefore, we examined whether the galactose elimination capacity (GEC), a physiological measure of the total metabolic capacity of the liver, was associated with mortality in a large cohort of patients with newly-diagnosed cirrhosis.

**Methods:**

By combining data from a GEC database with data from healthcare registries we identified cirrhosis patients with a GEC test at the time of cirrhosis diagnosis in 1992–2005. We divided the patients into 10 equal-sized groups according to GEC and calculated all-cause mortality as well as cirrhosis-related and not cirrhosis-related mortality for each group. Cox regression was used to adjust the association between GEC and all-cause mortality for confounding by age, gender and comorbidity, measured by the Charlson comorbidity index.

**Results:**

We included 781 patients, and 454 (58%) of them died during 2,617 years of follow-up. Among the 75% of patients with a decreased GEC (<1.75 mmol/min), GEC was a strong predictor of 30-day, 1-year, and 5-year mortality, and this could not be explained by confounding (crude hazard ratio for a 0.5 mmol/min GEC increase = 0.74, 95% CI 0.59–0.92; adjusted hazard ratio = 0.64, 95% CI 0.51–0.81). Further analyses showed that the association between GEC and mortality was identical for patients with alcoholic or non-alcoholic cirrhosis etiology, that it also existed among patients with comorbidity, and that GEC was only a predictor of cirrhosis-related mortality. Among the 25% of patients with a GEC in the normal range (≥ 1.75 mmol/min), GEC was only weakly associated with mortality (crude hazard ratio = 0.79, 95% CI 0.59–1.05; adjusted hazard ratio = 0.80, 95% CI 0.60–1.08).

**Conclusion:**

Among patients with newly-diagnosed cirrhosis and a decreased GEC, the GEC was a strong predictor of short- and long-term all-cause and cirrhosis-related mortality. These findings support the expectation that loss of liver function increases mortality.

## Background

Cirrhosis is a chronic liver disease with high mortality [1]. The condition implies a loss of liver function, and it is a fundamental assumption that the extent of this loss is important for survival. Still, little is known about the association between the prognosis of the patients and their liver function. Clinicians therefore often use scores based on standard blood chemistry tests and clinical signs of liver disease, such as the Child-Pugh score and the MELD-score, as prognostic indicators [2]. However, these scores reflect symptoms and complications of the liver disease, not the liver function as such.

The carbohydrate galactose is metabolized nearly exclusively in the liver, and the elimination rate at blood concentrations high enough to yield near-saturated enzymatic conversion, the galactose elimination capacity (GEC), is used as a quantitative measure of the metabolic capacity of the liver. GEC is assumed to reflect the liver's total capacity to serve the vitally important metabolic homeostasis of the organism [3,4].

A number of studies support the concept that the metabolic capacity of the liver, as measured with the GEC, is associated with mortality of cirrhosis patients: A prognostic value of the test was documented in patients with acute liver failure [5], and studies of cirrhosis patients found that the GEC added prognostic information beyond that obtained by standard blood chemistry tests [6-9], although this was not a consistent finding [10-12]. These studies, however, included only selected cirrhosis patients; comprised an insufficient number of patients (between 35 and 194); had relatively short observation time; and aimed to examine the utility of including the GEC in clinical prediction rules, not its association with mortality [6-12]. Thus the concept of an association between GEC and mortality of cirrhosis patients remains unproven despite its biologic plausibility. The association is best examined in a large cohort of cirrhosis patients with wide variation in GEC, and neither the inclusion criteria nor the analysis should include possible correlates of GEC, such as standard blood chemistry tests or clinical signs of liver failure [13]. Furthermore, an analysis of the association between GEC and mortality should adjust for age and gender because the GEC declines with age and is higher in men than in women [14-16], and it should also adjust for comorbidity that has a considerable impact on mortality [17]. We have available a sufficiently large and well-defined patient material to conduct such an analysis: 781 Danish patients with newly-diagnosed cirrhosis followed for up to 13 years.

## Methods

According to Danish law, studies that are based exclusively on data from administrative registries and clinical databases, such as this, require neither ethical approval nor patient consent.

### Study population

The study was based on GEC tests done between 1 August 1992 and 31 December 2005 in the two Danish tertiary referral centers for liver disease. All tests were performed as previously described [18,19]: A galactose solution is injected intravenously over 5 minutes (1 ml/kg body weight of a 500 mg/ml galactose solution), arterialized capillary blood is sampled every 5 minutes from 20 to 45 minutes to measure blood concentration of galactose, and urine is collected for 4 hours to measure urinary excretion of galactose. The GEC is calculated from the injected amount minus the excreted amount and from the straight line connecting the observations of blood galactose concentration against time:

where 'injected' is the amount of galactose administered, 'excreted' is the amount excreted in the urine, 'time_conc = o_' is the time at which the blood galactose concentration would theoretically reach zero, and 7 minutes a correction for delay of galactose equilibration between extra- and intravascular spaces.

The average GEC values in men and women without liver disease are 2.7 (95% CI 1.7–3.6) and 2.4 (95% CI 1.4–3.4) mmol/min, respectively [16]. No other quantitative liver function test was systematically performed.

We identified 3388 patients with a GEC test, among whom 781 had been diagnosed with cirrhosis less than 90 days before their first test, according to hospital discharge diagnoses recorded in the Danish National Patient Registry. This registry records individual-level information from all admissions to Danish hospitals since 1977, and from all outpatient visits since 1995 [20]. The information includes primary and secondary discharge diagnoses coded according to ICD8 (before 1994) or ICD10 (from 1994), and we defined cirrhosis by the following codes: 571.09, 571.92, 571.99, K70.3, and K74.6. Comorbid diseases were identified in the same registry and defined by the Charlson comorbidity index, based on 19 common chronic diseases, as previously described [17,21].

### Statistical analysis

The patients were followed from their first GEC test until death or 31 December 2005. Dates of death were obtained from the Danish Civil Registration System [22].

#### Cumulative mortality

The cumulative mortality was estimated as the complement of the Kaplan-Meier estimate of survival probability. We examined whether GEC was a predictor of mortality by dividing the patients into 10 equal-sized groups according to GEC (i.e., GEC-deciles) and plotting each decile's median GEC against its 30-day, 1-year, and 5-year mortality. We used lowess smoothing to facilitate the visual interpretation of the plots [23].

#### Cox proportional hazards regression

In order to examine whether the association between GEC and mortality was confounded, we estimated the hazard ratio associated with a 0.5 mmol/min GEC increase before and after adjustment for gender, age at GEC test, and presence of comorbidity (defined by a Charlson comorbidity index of 1 or higher). Based on the analysis of cumulative mortality we conducted separate analyses for patients with a GEC <1.75 mmol/min (decreased liver function) or ≥1.75 mmol/min. Among patients with a GEC below 1.75 mmol/min, the Cox regression model violated the assumption of proportional hazards, but this violation was of no consequence because the model still yielded the *average *hazard ratio during follow-up [24], and that was sufficient to examine the impact of confounding.

#### Cirrhosis-related mortality

In Denmark, cause(s) of death are immediately reported by the attending physician to the Danish Cause of Death Registry. We used data from the registry to compute the cumulative incidence of cirrhosis-related death and of death from other causes [25]. Cirrhosis-related death was defined as having cirrhosis, liver failure, or variceal bleeding as a cause of death; all other deaths were classified as not cirrhosis-related. In this analysis, cirrhosis patients were censored on 31 December 2001 because cause of death-registration was incomplete after 2001. The analyses were repeated with restriction to patients with alcoholic cirrhosis (diagnosis codes 571.09 or K70.3), non-alcoholic cirrhosis, or cirrhosis with comorbidity.

## Results

Among the 781 patients, 454 (58%) died during follow-up. The total observation time was 2,617 years with a median of 2.5 years per patient and a maximum of 13.3 years. The median age at inclusion was 52 years and 65% were men. GEC ranged from 0.59 to 3.97 mmol/min (median 1.48 mmol/min). The GEC was not associated with age, but it was higher in men than in women (median GEC 1.54 vs. 1.40 mmol/min) and in the 29% of patients with comorbidity (median GEC 1.57 vs. 1.45 mmol/min). The most prevalent comorbidities were diabetes (9% of patients at inclusion), ulcer disease (9%), and atherosclerosis-related disease (8%), which collectively comprised 78% of all comorbidity.

**Figure 1:**
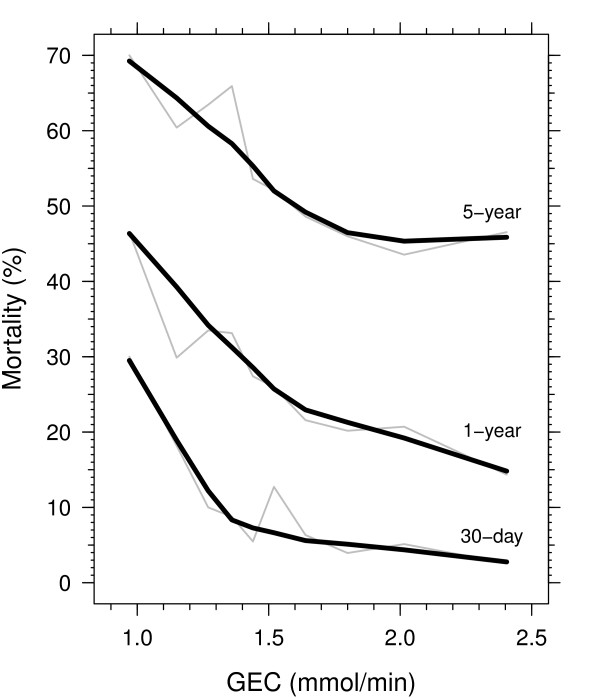
**All-cause mortality after 30 days, 1 year, and 5 years by GEC for the 781 included cirrhosis patients**. The gray lines connect each GEC-decile's median GEC with its observed mortality, and the black lines are lowess smoothings of the gray lines.

GEC was a strong predictor of short- and long-term mortality, particularly among the 75% of patients with a GEC below 1.75 mmol/min (Figure 1). For example, patients with a GEC of 1.75 mmol/min had a lower mortality than patients with a GEC of 1.25 mmol/min: 5% vs. 14% after 30 days, 22% vs. 35% after 1 year, and 48% vs. 61% after 5 years (Figure 1), and adjustment for potential confounders strengthened the GEC-mortality association (crude hazard ratio = 0.74, 95% CI 0.59–0.92; adjusted hazard ratio = 0.64, 95% CI 0.51–0.81), primarily because male gender was a risk factor for death and also associated with a high GEC (Table 1). Among the patients with a GEC above 1.75 mmol/min, the association between GEC and mortality was weaker and unaffected by confounding (crude hazard ratio = 0.79, 95% CI 0.59–1.05; adjusted hazard ratio = 0.80, 95% CI 0.60–1.08) (Figure 1 and Table 1).

**Table 1:** Impact of confounding by gender, age, and comorbidity on the association between GEC and mortality of cirrhosis patients, estimated with Cox proportional hazards regression.

		Crude HR (95% CI)	Adjusted HR (95% CI)
GEC < 1.75 mmol/min			
	GEC, per 0.5 mmol/min	0.74 (0.59–0.92)	0.64 (0.51–0.81)
	Male vs. female	1.31 (1.06–1.63)	1.41 (1.13–1.76)
	Age, per decade	1.22 (1.11–1.35)	1.20 (1.09–1.32)
	Comorbidity, CCI ≥ 1 vs. 0	1.40 (1.11–1.78)	1.37 (1.08–1.75)
GEC ≥ 1.75 mmol/min			
	GEC, per 0.5 mmol/min	0.79 (0.59–1.05)	0.80 (0.60–1.08)
	Male vs. female	1.46 (0.87–2.46)	1.44 (0.86–2.43)
	Age, per decade	1.28 (1.07–1.52)	1.23 (1.02–1.48)
	Comorbidity, CCI ≥ 1 vs. 0	1.59 (1.08–2.35)	1.42 (0.95–2.12)

Comorbidity is defined by the Charlson comorbidity index (CCI). Associations are expressed as hazard ratios (HR) and presented without adjustment (Crude HR) and with adjustment for the potential confounders.

The mortality was similar for patients with alcoholic cirrhosis (76% of patients) or non-alcoholic cirrhosis, and GEC had the same association with mortality in both groups. Furthermore, the GEC-mortality association was also present among the cirrhosis patients who had comorbidities.

**Figure 2:**
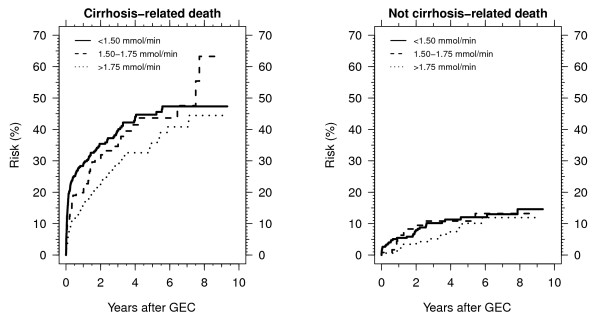
**Mortality from cirrhosis-related and not-cirrhosis related causes by GEC for the 591 cirrhosis patients who were included before 31 December 2001**.

Of the 587 patients who were available for the analysis of causes of death, 275 (47%) died during follow-up, 219 (80%) from cirrhosis-related causes and 56 (20%) from other causes. Irrespective of GEC, the risk of cirrhosis-related death exceeded the risk of death from other causes. The GEC was clearly a predictor of the risk of cirrhosis-related death, but not of the risk of death from other causes (Figure 2).

## Discussion

In this study of a large cohort of patients with newly-diagnosed cirrhosis and long-term follow-up we found that GEC was a predictor of mortality, also after adjustment for the effects of possible confounders, including comorbidity. As expectable, it was a stronger predictor of mortality among the 75% of patients with a decreased GEC (below 1.75 mmol/min) than among the 25% of patients with a well preserved or normal GEC.

Our findings are consistent with the existing studies of GEC and mortality of cirrhosis patients [6-12,26,27], but our study extends them by being sufficiently large to examine subgroups; by examining both cirrhosis-related mortality and mortality from other causes; by having complete long-term follow-up; by presenting a near-continuous relationship between GEC and short- and long-term mortality; and by adjusting for relevant confounders.

The dominance of alcoholic cirrhosis was expected [28], since viral hepatitis is not endemic in Denmark [29,30]. Patients with alcoholic liver disease are admitted to hospital when they are ill, whereas patients with viral hepatitis are followed in outpatient surveillance programs; therefore patients with alcoholic cirrhosis are more likely to have advanced cirrhosis at the time of diagnosis. This may explain why 75% of our patients had a decreased liver function at the time of cirrhosis diagnosis. This characteristic of our cohort should not detract from the generalizability of our findings, since we found the same association between GEC and mortality in patients with alcoholic and non-alcoholic cirrhosis.

We did not use clinical and laboratory data in the present study because such data would primarily have been used to substantiate the diagnosis of cirrhosis. Rather, we used the final diagnoses reported to the National Patient Registry. On a nationwide basis, about 15% of the cirrhosis diagnoses in this registry are wrong [31], but we expect this percentage to be markedly lower in our study because all patients were recruited from referral centers for liver disease with focused diagnostic work-up programs; the large proportion of cirrhosis-related deaths supported this assumption.

In our analyses we adjusted for gender, age, and comorbidity, but we cannot rule out that other confounders may have contributed to the GEC-mortality association. However, no confounder can realistically explain the full extent of the association.

The GEC test has not gained widespread use in clinical hepatology despite its advantage of measuring the liver's metabolic capacity. Likely reasons include the need for intravenous administration of galactose, sampling of capillary blood, and subsequent laboratory work, as well as an inherent individual variability in test results of up to 10–20% by repeated measurements [32], partially due to variation in the time course of GEC among patients [33]. Additionally, it is still uncertain whether GEC improves the accuracy of clinical prediction rules based on clinical data and standard blood chemistry tests to an extent that justifies its complexity [6-12]. The present findings, however, indicate that GEC may be able to transcend clinical features of cirrhosis and deliver unconfounded and prognostically meaningful information on liver function. When such robust information is important, e.g. in critical clinical situations involving patients with multiple complications and comorbid diseases, the GEC may present a unique basis for decisions, its inconveniences notwithstanding.

## Conclusion

Our study demonstrated the association between GEC measured at the time of cirrhosis diagnosis and all-cause and cirrhosis-related short- and long-term mortality. GEC was a strong predictor of mortality among patients with a decreased GEC, below 1.75 mmol/min, and a weak predictor of mortality among patients with a higher GEC. These findings are in accordance with the concept that the total metabolic capacity of the liver is important for the prognosis of patients with chronic liver disease.

## Competing interests

The authors declare that they have no competing interests.

## Authors' contributions

PJ, HV, and NT conceived and designed the study. PJ and PKA analyzed the data, and all authors interpreted the data. PJ and HV drafted the manuscript, and PO, SK, PKA, and NT revised it. All authors read and approved the final manuscript.

## Pre-publication history

The pre-publication history for this paper can be accessed here:

http://www.biomedcentral.com/1471-230X/9/50/prepub
